# MRI-LINAC: A transformative technology in radiation oncology

**DOI:** 10.3389/fonc.2023.1117874

**Published:** 2023-01-27

**Authors:** John Ng, Fabiana Gregucci, Ryan T. Pennell, Himanshu Nagar, Encouse B. Golden, Jonathan P. S. Knisely, Nicholas J. Sanfilippo, Silvia C. Formenti

**Affiliations:** ^1^ Department of Radiation Oncology, Weill Cornell Medicine, New York, NY, United States; ^2^ Department of Radiation Oncology, Miulli General Regional Hospital, Acquaviva delle Fonti, Bari, Italy

**Keywords:** MRI, external beam radiotherapy, radiation therapy technology, image-guided radiation therapy, MR-guided radiation therapy, medical physics

## Abstract

Advances in radiotherapy technologies have enabled more precise target guidance, improved treatment verification, and greater control and versatility in radiation delivery. Amongst the recent novel technologies, Magnetic Resonance Imaging (MRI) guided radiotherapy (MRgRT) may hold the greatest potential to improve the therapeutic gains of image-guided delivery of radiation dose. The ability of the MRI linear accelerator (LINAC) to image tumors and organs with on-table MRI, to manage organ motion and dose delivery in real-time, and to adapt the radiotherapy plan on the day of treatment while the patient is on the table are major advances relative to current conventional radiation treatments. These advanced techniques demand efficient coordination and communication between members of the treatment team. MRgRT could fundamentally transform the radiotherapy delivery process within radiation oncology centers through the reorganization of the patient and treatment team workflow process. However, the MRgRT technology currently is limited by accessibility due to the cost of capital investment and the time and personnel allocation needed for each fractional treatment and the unclear clinical benefit compared to conventional radiotherapy platforms. As the technology evolves and becomes more widely available, we present the case that MRgRT has the potential to become a widely utilized treatment platform and transform the radiation oncology treatment process just as earlier disruptive radiation therapy technologies have done.

## 1 Introduction

The development of a linear accelerator (LINAC) system with an integrated Magnetic Resonance Imaging (MRI) scanner is a major advance in image guided radiation technology (1, 2). Previously, image guided radiation therapy would rely on on-board portal film imaging or planar kV radiographs or cone-beam computed tomography (CT) scanning during patient setup for image verification before radiation dose delivery. With the integration of an on-board MR scanner within the LINAC radiation therapy system, real time image guidance throughout tumor and organ motion and during radiation delivery became feasible.

In the field of external beam photon radiation oncology, several earlier technical and technological improvements - including intensity-modulated radiotherapy (IMRT), volumetric arc radiotherapy (VMAT) and stereotactic radiotherapy (SRT) - have been implemented in daily clinical practice. A point of crucial interest with these new technologies has been the faculty to control, verify, and eventually modify the treatment planning and delivery process with high accuracy and precision. This power to achieve a more homogeneous better target volume coverage is coupled with the ability to significantly reduce the volume of healthy tissue irradiated to high doses (3).

These great efforts to push the boundaries of a safe and effective radiotherapy plan have raised a central question - “how can we know during treatment delivery whether what we have so carefully measured and calculated in the planning process is the actually delivered treatment?” In fact, the central challenge of precision radiotherapy remains the intra-fraction variability of the target, i.e., controlling for the individual physiological body movements at the precise moment of dose delivery.

To address this key challenge, several solutions have been brought forth, leading to the revolutionary concepts of tumor tracking and adaptive radiotherapy (ART). Real time motion management and ART reduces the uncertainties related to imaging, treatment planning, and treatment delivery due to daily organ variability and motion, tumor delineation (including microscopic disease) and inter- and intra- fraction setup error and variability. Historically, the generation of significant planning target volume (PTV) margins around the target was introduced to compensate for these uncertainties, with the drawback of irradiating more healthy tissue and increasing the toxicity to organs at risk (OaRs) (4). The development of 4-dimensional CT (4D-CT) allowed tracking of the tumor through the study and control of respiratory motion (5). 4D-CT was a major advance allowing the radiation oncologist and treatment team to reduce PTV margins related to motion during the respiratory cycle. However, organ motion control and verification remain complex problems requiring different solutions. Target uncertainty not only depends on breathing but it is also associated with motion/changes in other organs such as bowel peristalsis, bladder filling, and day-to-day anatomic variation. Hence, 4D-CT can neither resolve the issue of correct target definition nor account for inter- and intra-fractional anatomic changes.

In this era of highly customized and personalized therapy, a broader concept of adaptive image-guided and biology-guided RT has arisen (6–8). The field of image-guided radiation therapy interfaces thus intersects with the fields of radiomics and bioinformatics, including machine learning and artificial intelligence (AI) (9). We summarize three seminal applications of adaptive image-guided RT: i) therapy guidance (target and OaRs definition); ii) treatment plan verification (inter-fraction management); and iii) real-time delivery control (intra-fraction management).

### 1.1 Therapy guidance

The starting point of the care pathway in RT is represented by target volume and OaRs definition within the individual patient. The clinical team must accurately evaluate the area to be treated with a curative radiation dose and its relative anatomic relationships with the surrounding healthy tissue, to plan for a dose delivery that spares normal tissue as much as possible from irradiation. Kilovoltage CT imaging acquired at simulation is the standard basis for the construction of the treatment plan. It allows for morphological mapping of the anatomy based upon the distribution of differing electronic densities of various tissues. Today, highly sophisticated and complementary imaging modalities, such as MRI and positron emission tomography (PET) are merged with the CT simulation images. Fusion with these additional imaging modalities lead to a better morphological and structural definition of the area being treated as well as the integration of metabolic and functional information (10–12). Fusion occurs in the planning phase but lack of advanced on-board imaging within the standard linear accelerator precludes a precise application for each dose delivered.

### 1.2 Treatment verification

Another critical aspect of radiation delivery which relies upon image guidance is treatment verification and the possibility of real-time re-planning in case of anatomical variations related to disease response or to human physiology that may occur during the course of RT. In the past, the best available on board imaging technique was 2D radiological imaging obtained using low contrast MV or kV x-rays which permitted visualization of bony landmarks or suitably positioned radiopaque markers to verify target coverage (13).

Daily transabdominal ultrasonic spatial localization of the prostate is an example of a non-invasive approach that avoided radio-opaque fiducial markers implantation’s expense, discomfort, and risks (14). The introduction of cone beam CT (CBCT) technology has permitted volumetric visualization of the anatomical treatment field, improving accuracy in management of inter-fraction variations. In some clinical sites such as brain, abdomen and pelvis, however, CBCT imaging does not allow sufficient definition of the soft tissue, often burdened by significant artifacts from the presence of air and scattered photons that limit imaging accuracy of cone beam CT reconstruction algorithms (15).

Surface matching algorithms are another approach that has recently been used for guiding radiotherapy treatments. Surface matching, however, does not contain direction information about the location of the target volume or organs at risk that are not immediately correlated to the surface markers.

These treatment verification strategies were developed for treatment courses where a radiotherapy treatment was planned once. Every effort would be exerted to provide reproducible geometries and additional margins would be introduced during planning to assure that the treatment would not miss the target. Fractionation schemes were selected to permit normal tissue included in the high dose volume to not exceed established acute or long-term tolerances.

As technologies improved to precisely deliver highly conformal intensity modulated treatments and technologies emerged to assess the relationships of target organs to organs at risk, it was realized that a radiation plan generated from a remotely acquired imaging study may not reflect the optimal treatment plan on the day of delivery. The ability to acquire imaging, plan treatment, and deliver treatment using real-time image guidance, which an MR-guided linear accelerator is capable of, breaks through multiple barriers to providing better care.

### 1.3 Delivery control

A major goal of image guided radiotherapy is the possibility to see in *real time* what happens at the treatment site during the delivery phase and to intervene/adjust if there is a significant shift on the target. This challenge is the latest frontier of adaptive guided-RT application, and reinforces the need for technologies which can address intra-fraction motion management and pave the way for safe dose escalation and de-escalation therapy (6).

A current standard for accounting for real time tumor and organ motion is four-dimensional computed tomography (4D-CT). 4D-CT utilizes a set of CT images acquired throughout different phases of the patient’s respiratory cycle and combines them with tracking of external respiratory markers during patient setup and delivery. The uncertainties associated with 4D-CT are accounted for by the expansion of internal target volume (ITV) and planning target volume (PTV) margins, theoretically compensating for intra-fractional tumor and organ motion. The individual breathing cycle is studied during simulation and dose delivery is planned consistently.

However, 4D-CT is inherently limited by the daily reproducibility of the breathing cycles and does not control for changes in daily tumor and organ motion (16). In other words, 4D-CT informs a plan to improve delivery control and motion management through images acquired during CT simulation, but it cannot represent real-time, daily motion management. Ultimately, representative delivery control is possible only with real-time motion visualization through on-board intra-fraction imaging, target structure tracking, and gated treatment delivery.

We present the case that MRgRT is the most promising disruptive radiation oncology technology to overcome the challenges of intra-fraction motion. Through improving upon contemporary image-guided radiation technology, MRgRT is transforming the radiotherapy delivery process within radiation oncology centers, reorganizing patient flow and how treatment team members interact. In this review, we describe some of the advantages that MRgRT provides and the remaining major barriers to its routine adoption. We summarize the original data in disease sites where MRgRT has already had an impact. Finally, we introduce some emerging developments involving MRgRT.

## 2 MRI-Linac systems and other platforms

In recent years, several radiotherapy platforms have become commercially available in clinical radiation oncology to meet the challenges of adaptive image-guided RT. Similar to the earlier technical advances described above, these newer technologies are starting off as resource intensive approaches with specialized clinical applications. With continuous stepwise improvements, the reduced toxicity and other clinical advantages made possible by IMRT, VMAT, and stereotactic RT overcame the initial barriers of cost and resource investment (6, 17). It is expected that over time, incremental improvements and broader indications will enable these newer technologies to be widely disseminated into standard radiation oncology practice.

There are currently two main MRgRT platforms commercially available – the ViewRay MRIdian system (Viewray Inc., Oakwood, OH) which uses a 0.35 Tesla MRI scanner and the Elekta Unity (Elekta AB, Stockholm, Sweden) system which uses a 1.5 Tesla MRI scanner. The ViewRay system initially used cobalt-60 as its radiation source and received FDA approval in 2012 (18). ViewRay then developed a platform where the MRI scanner was integrated within a linear accelerator, receiving FDA clearance in 2017 (19). The Elekta Unity system was approved in 2019 by the FDA as the second MRI-linear accelerator system (20).

More recently, other hybrid linear accelerator systems with adaptive capabilities have also gained FDA clearance and are treating patients in the clinic. Varian’s Ethos system (Varian, Palo Alto, CA), FDA cleared in 2020, enables adaptive radiotherapy utilizing on-board fan-beam CT imaging (21). The most recent is the Reflexion system (RefleXion, Hayward, CA), a radiotherapy system that the FDA approved in 2021 with future plans to utilize an on-board PET scanner as the integrated imaging modality used for guidance while treating patients on a linear accelerator (22). The Ethos and the Reflexion systems join the earlier Cyberknife system (Accuray, Sunnyvale, CA) and Radixact system (Accuray, Sunnyvale, CA) as non-MRgRT based adaptive radiotherapy systems. All these radiotherapy technologies revolve around the idea that future radiation oncology practices will leverage the ability to acquire imaging while the patient is on the table to account for motion management and adapt treatment planning on the day of delivery. The Viewray MRIdian has the faculty of real time intrafraction modulation of dose delivery by imaging during treatment delivery, and the Elekta Unity scanner introduced support for motion management during radiation delivery in October of 2022.

**Table 1** summarizes the features of some of the most common commercially available IGRT platforms that have received FDA regulatory clearance for radiation delivery in patients.

**Table 1:** Commercially available radiotherapy platforms specializing in adaptive image-guided and biology-guided radiation therapy.

System	Image guided modality	Real-timeinter-fraction management	Real-timeintra-fraction management	Strengths	Weaknesses
**MRIdian**	MR (0.35 Tesla)	Yes	Yes, automatically	No ionizing radiations for imaging	Time
				Target visualization during treatment	Coplanar beam fields
				High soft tissue discrimination	No electronic density data
				Functional imaging data	
**Unity**	MR (1.5 Tesla)	Yes	Yes, not automatically	No ionizing radiations for imaging	Time
				Target visualization during treatment	Coplanar beam fields
				High soft tissue discrimination	No electronic density data
				Functional imaging data	
**Ethos**	Artificial Intelligence CT based	Yes	No	Time	Use of ionizing radiations for imaging
					Coplanar beam fields
				Electronic density data	No target visualization during treatment
					Low soft tissue discrimination
					Functional imaging data
**RefleXion**	PET-CT	Yes	No	Time	Use of ionizing radiations for imaging
				Electronic density data	Coplanar beam fields
				Functional imaging data	No target visualization during treatment
					Low soft tissue discrimination

## 3 Main differences between the MRI-Linac-based systems

Between the two widely utilized MRI-Linac systems, there are key differences in design and treatment features. We summarize them here and in **Table 2**:

**Table 2:** Comparison of the MRIdian and Unity MRI-Linac radiotherapy platforms.

Feature	MRldian	Unity
**Construction**	Split Magnet Design	Single Magnet Design
**Imaging**	Trufi Sequence Imaging based	Range of Imaging sequences available
**Gating**	Real time tracking and automatic gating	Real time tracking without automatic gating
**Treatment**	Gantry rotation maximum speed of 0.5 rpm	Gantry rotation maximum speed of 6.0 rpm

### 3.1 Construction

MRIdian: The split superconductor 0.35 Tesla magnet design allows for a smaller source-to-axis distance (SAD). The integration of the linear accelerator and the magnet allows for robust integration of imaging registration with treatment planning capabilities.

Unity: A single magnet design with the LINAC components placed outside the 1.5 Tesla MR scanner. Due to its greater magnet field strength with this design allows for greater imaging capability compared to a lower-field imaging system.

### 3.2 Treatment delivery

MRIdian: The MRIdian utilizes coplanar static IMRT fields and can deliver radiation dose at a 650MU/min dose rate. The gantry rotation speed is 0.5 rpm with no collimator rotation.

Unity: The Unity utilizes coplanar static IMRT fields and can deliver radiation dose at a 500MU/min dose rate. The gantry rotation speed is 6.0 rpm gantry with no collimator rotation.

### 3.3 Imaging

MRIdian: The MRIdian utilizes a balanced steady state free precession (SSFP) MRI pulse sequence for planning, setup, and treatment delivery. Other MRI pulse sequences, such as T2/T1 and DWI sequences, can only be used as registered images alongside the balanced SSFP sequence.

Unity: The Unity has a broad range of pulse sequences available for planning and treatment. MR imaging is available during the treatment. Up to recently, if the clinical team chooses to image with the MRI host during treatment, they would lose the ability to track the target during delivery. Very recently, Elekta has introduced a motion management package that overcomes this limitation.

### 3.4 Gating

MRIdian: The MRIdian is able to automatically gate on one sagittal slice delineated from the 3D volumetric scan at 8 frames per second. Its newer A3I features allow for tracking capabilities on all three planes (sagittal, coronal, and axial) simultaneously or on multiple planes in the same orientation. The beam will automatically gate itself once the target migrates too far beyond the defined boundary expansion.

Unity: The Unity has the ability to track targets in real time on three planes (sagittal, coronal and axial). If the target moves outside the pre-specified envelope, the treatment beam will be automatically gated by the machine (currently pending FDA approval, CE Marked in the European Union).

## 4 Advantages of the MRI-Linac Technology

**Figure 1:**
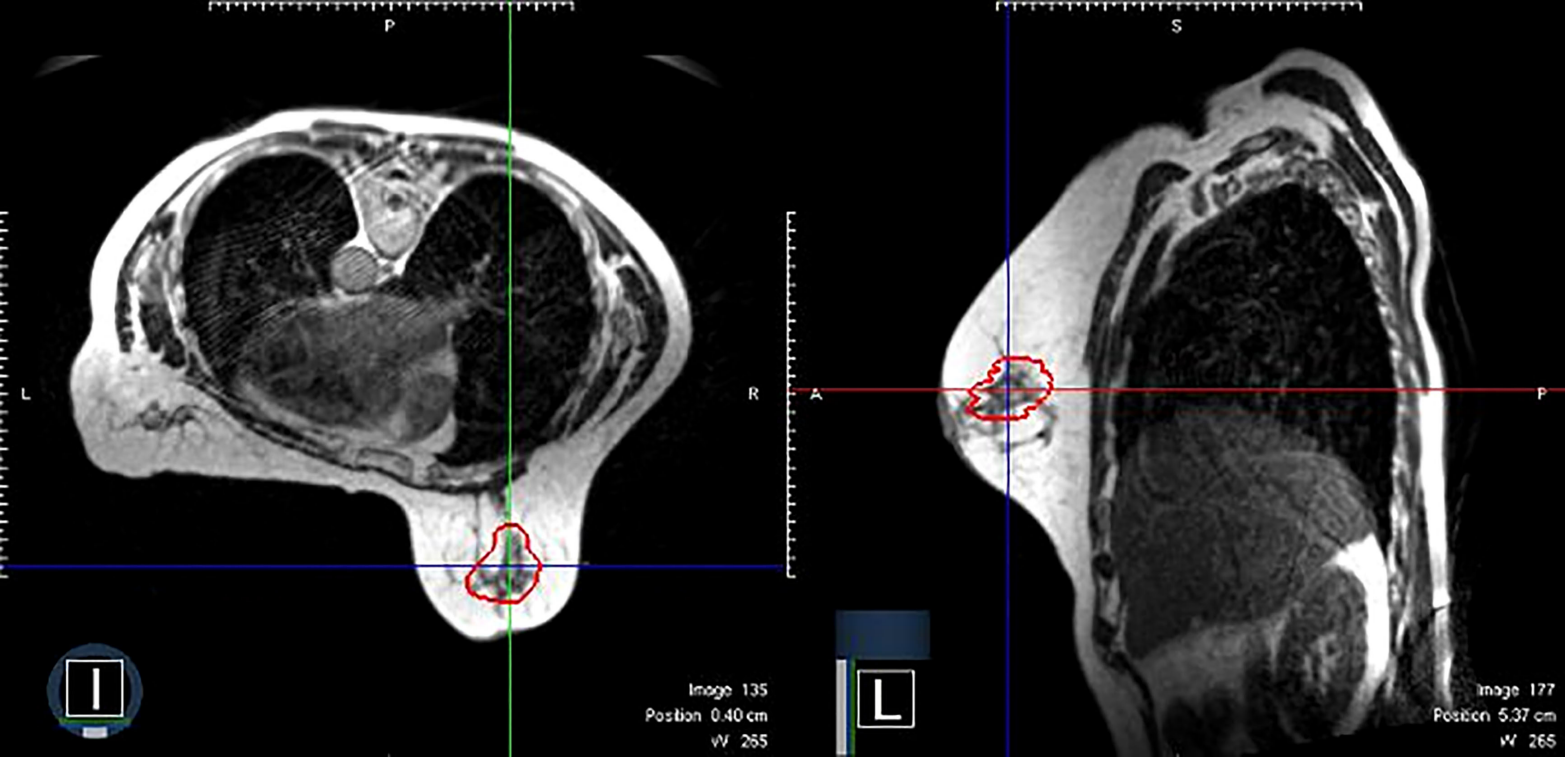
An example of clinical MRI-Linac images utilized for therapy guidance. The panel on the left shows a patient receiving prone breast irradiation with an MRI image in the axial plane. The same patient with a sagittal plane image. The red contour depicts the lumpectomy surgical cavity which serves as the clinical target volume.

**Figure 2:**
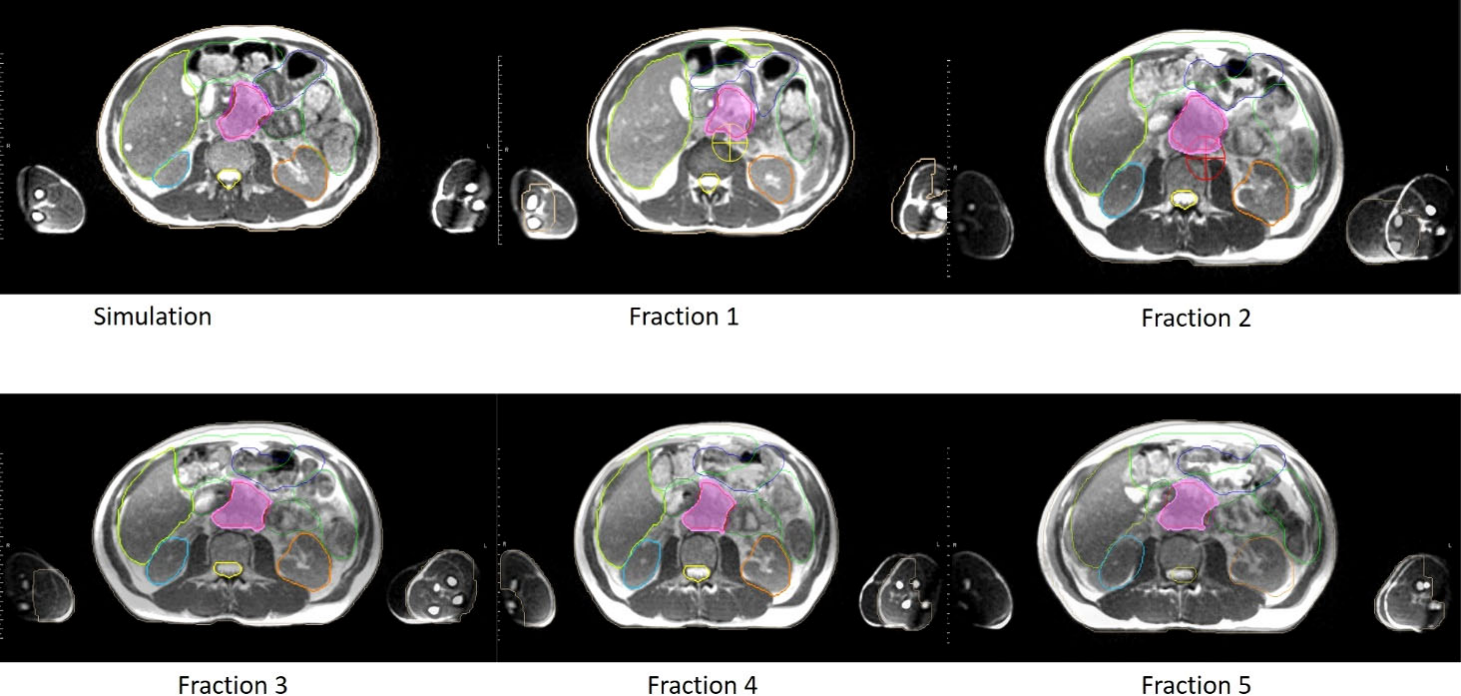
An example of clinical MRI-Linac images utilized for inter-fractional management. Comparison of anatomy seen on the day of simulation (upper left panel) and days of treatment (panels labeled Fractions 1 to 5 respectively) for a pancreatic cancer patient treated at our institution. In each panel, the target volume, stomach, and bowel anatomy are contoured.

**Figure 3:**
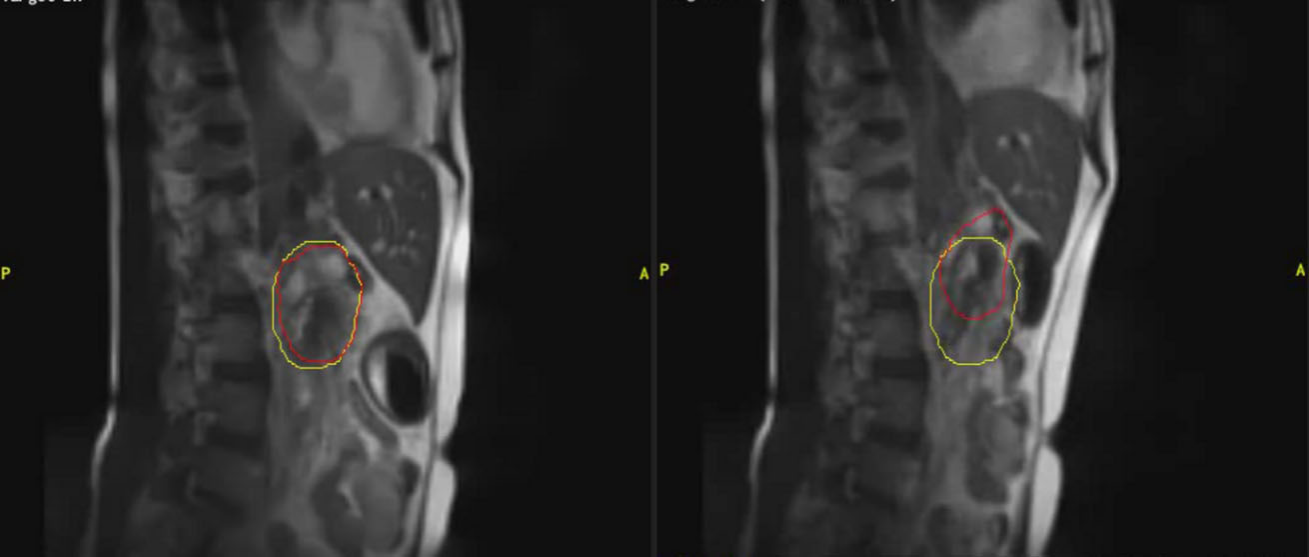
An example of clinical MRI-Linac images utilized for intra-fractional management. The panel on the left shows a patient receiving pancreatic radiotherapy while imaged with a deep inspiratory breathe hold. The panel of the right shows the same patient breathing freely. The red contour depicts the tracking contour and the yellow contour depicts the treatment envelope boundary.

To address the challenges posed by image guided radiation therapy at the present, we will review three key advances in adaptive image-guided RT made possible by the MRI-Linac technology: 1) imaging for therapy guidance (**Figure 1**); 2) adaptive treatment planning for inter-fractional management (**Figure 2**); and 3) real time imaging and gating for intra-fractional management (**Figure 3**).

### 4.1 MRgRT: Imaging for therapy guidance

MR imaging is an imaging technique based on nuclear magnetic resonance which maps the spatial concentration of signal bearing spins of the tissue environment. The imaging is also dependent on differing signal intensities between the tissues. These properties permit MR images to have higher resolution relative to CT imaging, even without the administration of contrast. A range of possible MR pulse sequences that can be used clinically can allow the acquisition of different forms of images, most commonly T1-weighted, T2-weighted or proton density based imaging sequences, each characterized by a different signal intensity that creates contrast between the various tissues. In T1-weighted images, fluid is hypointense and fat is hyperintense while in T2-weighted images the fluid is hyperintense and the fat is mildly hypointense (23). The features of T1- and T2-weighted imaging are often used in RT for the anatomical definition of the target and the OaRs due to their signal contrast in soft tissue (24). There is further promise that combining different MR pulse sequences, such as combining different functional spin echo-based and/or gradient echo-based sequences, can allow MR imaging to obtain different functional information that could help characterize the tumor microenvironment. These sequences could include fluid-attenuation inversion recovery (FLAIR), short tau inversion recovery (STIR), diffusion-weighted imaging (DWI) and dynamic contrast-enhanced (DCE) (25). As MR image voxels can measure quantitative properties over time, they allow measurements of parameters that are indicators of tumor cell density (with DWI), vascularity (with perfusion), stiffness/stroma (with elastography) and hypoxia (with relaxometry), biologic factors that are known to drive radiosensitivity and radioresistance (26, 27). Overall, MR imaging is more versatile and can potentially unlock addition clinical information complementary to conventional CT imaging.

### 4.2 MRgRT: Adaptive treatment planning for inter-fractional management

The anatomic changes that occur from the time of simulation to when daily treatments are initiated, and in between daily treatments have been a problem in treatment verification, often necessitating wider target margins to ensure the target received full dose radiation delivery. This is often counterbalanced by limiting the total prescription dose and coverage to limit the risk of toxicity. The current standard 4D-CT involving CT imaging, sometimes with the placement of fiducial markers, at the simulation and in the treatment room throughout the respiratory cycle before delivery was a major advance in the field, but there remained the persistent issue of adjusting for these day-to-day positional changes in the tumor target and OaRs (28). For example, several studies of abdominal tumors and organs showed that the daily variation of the pancreas position could exceed 1 cm in each direction (29, 30).

Adaptive radiation treatment planning (ART) is an approach that allows for daily adjustments of the radiation treatment plan based on re-determining spatial parameters for the treatment targets and nearby tissues. While this can be occasionally done on acquired images with the patient off the table, a significant step forward would be performing the same tasks while the patient is on the treatment table. Conventional linear accelerators are not capable of such an approach.

The advent of the hybrid MRI-Linac systems make routine clinical implementation of on table ART possible. The MRI-Linac systems feature full integration of the treatment planning software with the radiation delivery unit and also feature newer rapid dose calculation algorithms. Adaptive re-planning of radiation treatments with MRI guidance have now been shown to be feasible and to offer comparable plan qualities to their respective reference treatment plans (31). The ability to acquire an updated MRI scan while the patient is on the treatment table, to adjust for anatomic changes prior to radiation delivery, and to adapt the treatment plan could allow for tighter margins on treatment volumes and enable dose escalation with favorable toxicity when compared to non-adaptive radiation planning, but prospective clinical data will be necessary to establish these benefits.

### 4.3 MRgRT: Real time imaging and gating for intra-fractional management

As discussed above, current motion management strategies such as 4D-CT are passive, utilizing patterned or expected motion to determine additional safety margins such as an ITV. An improved intra-fractional management strategy would be active, such as beam gating, whereby a pre-specified target is monitored in motion and the beam is turned on only when that target is within a pre-defined window.

For beam gating, the hybrid MRI-Linac systems have the distinct advantage of real-time imaging of soft tissues (32). This major technical advance enables the clinical team to visualize with high accuracy the target during overall treatment course and provides the ability to monitor in real time the physiologic moments of internal organs that impact on intra-fraction reproducibility of dose delivery.

With recent upgrades on the Unity, both MRI-Linac systems are now capable of *automatic*, active intra-fractional beam gating. With the MRIdian system, two-dimensional cine MRI images are acquired in the sagittal plane (can also be done in the axial and coronal planes) at 8 frames per second. A gating tracking target is contoured during a breath-hold MR scan, and a gating envelope is generated off a margin expansion from that contoured gating target. As radiation treatment is being delivered, if a pre-specified volume (for example, 5%) of the gating target is outside the gating envelope, the beam is automatically turned off. If the total volume of the gating target falls within the gating envelope by a defined threshold, the beam automatically turns on. Later in this review, we will describe several disease sites where automatic real-time gating may have powerful clinical applications.

MRgRT permits the clinical team to set threshold boundaries on the maximum safe displacement of the treatment target (33). In turn, real time intra-fractional imaging and active gating provide the possibility of reducing treatment margins, of increasing target dose, and of sparing dose to organs at risk (OARs) (34). The approach is applicable to many clinical scenarios including thoracic, abdominal and pelvic disease. In those anatomic regions, the proximity and movement of OaRs limit target dose, and dose escalation may improve oncologic outcomes and dose avoidance may reduce toxicity (35).

Compared to conventional on-board imaging of a CT-based linear accelerator, MRgRT does not use ionizing radiation to obtain real-time images. This is a great advantage in terms of patient protection, but it has fostered a new argument relative to the effect on human body of long exposure to radiofrequency energy emitted by a MR scanner (28). This energy causes heating of the body in a proportional way to the square of the magnetic field strength and it may be a future challenge to understand its potential implications, particularly for individuals with implanted devices that may be more susceptible to localized heating (36).

## 5 Limitations of the MRI-LINAC technology

### 5.1 Capital costs

The most immediate barrier to wide implementation of the MRI-LINAC technology in radiation oncology centers are the capital costs associated with acquiring and maintaining the system. The current estimates are that initial acquisition price would be greater than $7.5 million for a MRI-Linac (37, 38). In addition, the annual maintenance costs are estimated to be greater than $500,000 per year.

Further initial expenses include construction and installation costs for a dedicated vault for a MRI-Linac with the necessary shielding and increased needed capacity for a superconducting magnet with helium, estimated to be greater than $14,000 per square meter (37).

### 5.2 Duration of treatment and throughput

A second major limitation is the time allocation needed for MRgRT treatments. A typical MRI-guided treatment requires daily patient checks to ensure MRI-capability. The patient then has to be set up according to simulation with appropriate MR coils placement. An MRI sequence is needed to be acquired for patient setup verification with physician approval. Finally, a tracking contour and a tracking cine has to be generated for treatment verification. In summary, the dedicated time to apply a real time treatment management workflow is a process that requires around 30-60 minutes for each treatment session (19, 39, 40). Two prospective phase I trials that included adaptive treatments in the thorax and in the abdomen on the MRI-Linac failed to meet their initially allotted fixed time constraint endpoints (41, 42).

**Figure 4:**
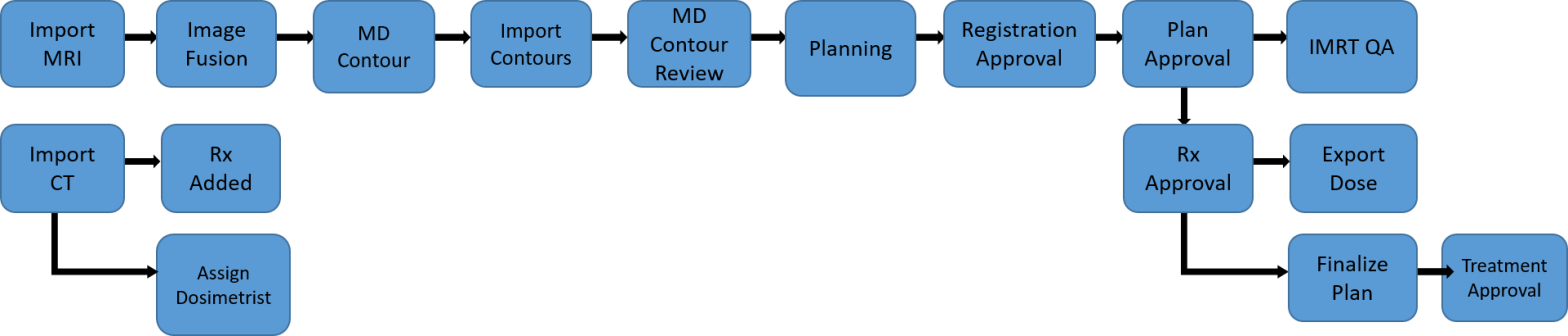
A representative example of the care pathway implemented during a MRgRT treatment. Each blue box represents a distinct task that a clinical treatment team member must complete before triggering the next task to be done, demonstrated by the arrow diagram.

For an adaptive treatment, further time must be allocated for each treatment. While the patient is on the table, the MRI image acquired is then utilized to predict the coverage and OAR doses from the pre-set treatment plan. If the predicted dosimetry is considered inadequate, the physician can re-contour the target volumes and the proximate OaRs. The treatment planning team with the physician, therapists, dosimetrist, and physicist at the planning console then creates a day of treatment plan. An optimized plan created on the day of treatment is approved and then delivered. The dedicated time for patients receiving an MRgRT treatment with ART would be around 90-120 minutes with all clinical team members present at the console and completing their individual tasks in close coordination (40) (**Figure 4**). There is further time complexity involved in the latency between the image acquisition and consequent action decision (43). Several algorithms have been proposed to mitigate this issue. Recently, Jöhl and colleagues elaborated a linear methods approach to predict the target displacement and proposed that hereafter artificial neural networks could be implemented (43, 44).

Given these time and resources allocated for MRgRT, the patient throughput with this technology is slower than that of a conventional linear accelerator (37, 39). Implementing a MRgRT program requires high quality imaging and an efficient workflow process to acquire the MRI scan, adapt the radiation plan, perform quality assurance, and deliver the radiation while the patient is set up for treatment. Wider practical applicability of MRgRT will be limited by the intense utilization of personnel and time to safely and properly deliver adaptive treatments, but exciting future developments are expected in the coming years which we will describe later.

### 5.3 Lack of non-coplanar beam delivery and other delivery limitations

While treatment delivery is more versatile in terms of intra-fractional and inter-fractional management with the MRI-Linac system, radiation delivery is also limited by the hybrid integration of the MR scanner within the linear accelerator. For example, it currently is not possible to deliver arc therapy or non-coplanar beam therapy with a MRI-Linac system (27). The current MRI-Linac systems also cannot deliver electron beam therapy (27).

Other delivery limitations are due to the design of the current MRI-Linac systems. When a patient is lying within the treatment bore, the ability to shift the table position or to rotate the couch is limited. Finally the size of the MRI bore limits its use to patients with an appropriate body habitus.

### 5.4 Contraindications to MR imaging

The MRI technology also has other established practical limitations and patient contraindications. Claustrophobia in MRI scanners are common in the general population. Some studies estimate that 10-15% of patients would require some level of sedation to be able to be able to complete an MRI scan (45). Hospitals and clinics have established safety policies and standards in place for MRI safety, and the clinical team must maintain vigilance to ensure that the patient does not have MRI-incompatible material in their body or on their person (46). Implanted medical devices such as pacemakers and defibrillators have to be interrogated before and after approaching an MRI scanner (47).

## 6 Disease sites and clinical applications

Despite the challenges just described that counterbalance wide adoption of MRgRT, acquisition of the technology and its utilization in the clinic has been steadily increasing. We will describe several disease sites where current clinical evidence justify their utilization and reinforce their unrealized potential.

### 6.1 Prostate cancer

MRI is routinely employed for prostate cancer diagnosis, staging, and management (48, 49), enabling identification of malignant portions within the prostate, as well as enhanced discrimination of the adjacent bowel, rectum and bladder (50). MRI is used as an adjunct imaging modality to CT-based radiotherapy planning to further delineate soft tissues (51). Additionally, MRI can aid with sparing the neurovascular structures associated with erectile dysfunction (52). However, static imaging acquired prior to treatment fails to capture changes in target volumes after the initial planning, as well physiological movement of internal organs (53–55). In contrast, MRgRT allows monitoring of both tumor changes and daily positional changes of internal organs for each treatment to achieve a more accurate estimation of a treatment plan’s dose distribution (i.e. inter-fraction adaptive planning), as well as real-time motion monitoring during treatment (i.e. intra-fraction gating) (56).

**Figure 5:**
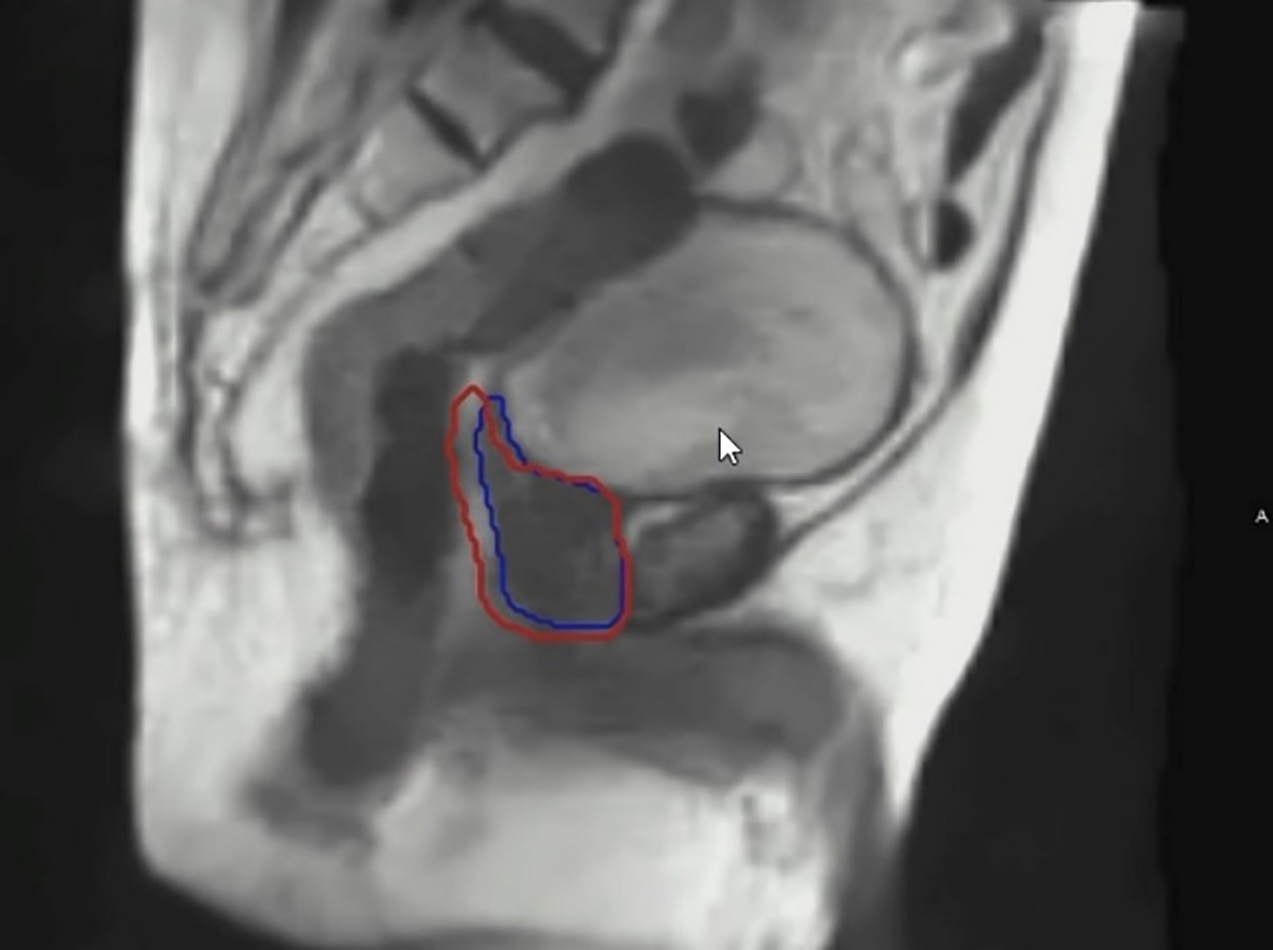
An example of real time imaging during a prostate MRgRT treatment. The blue contour depicts the prostate target tracking contour. The red contour depicts the gated treatment envelope boundary.

Adaptive planning utilizes day-of-treatment imaging and re-contouring of target volumes and/or organs at risk based on changes in their size or relative position. This can improve the therapeutic index in rapidly changing tumors, or in regions where there are dramatic changes in organ position during each fraction, such as the rectum for prostate treatment. Gating refers to synchronizing the radiation beam with a predetermined parameter. In respiratory gating, radiation is delivered within a specified range of respiratory motion so that the beam is turned off when there are deviations outside set inspiratory and expiratory parameters. An inherent benefit of MR is that there is no additional radiation exposure as a consequence of real-time imaging, and thus can be performed continuously during treatment delivery. In MR-guided therapy where real-time imaging is employed, gating of the target volume within a user-specified boundary is typically performed, in which the beam is only on if the PTV falls within the pre-defined boundary (see **Figure 5**).

In prostate radiotherapy, image gating is useful to account for organ movement resulting from bowel gas, stool passage and bladder filling as well as contracting of the muscles of the pelvic floor (57). Adaptive planning and gating are complementary techniques, in which adaptive planning corrects for inter-fraction anatomic variation and gating accounts for real-time, intra-fraction physiologic motion. Other potential roles for the MR-Linac in prostate cancer treatment include its applications for dominant lesion boosting and prostate re-irradiation (58, 59). Furthermore, intra-fractional motion management with MRgRT now enables enhanced ability to observe intra-fraction prostate motion during prostate SBRT (60, 61).

### 6.2 Lung cancer

Recent advances in lung cancer radiation therapy include stereotactic body radiation therapy (SBRT) for early-stage disease and IMRT for locally advanced cases (62). In either clinical situation, precise dose delivery is paramount to maximize local control and avoid toxicity.

While SBRT and IMRT have improved the therapeutic ratio, challenges still exist. In patients treated with SBRT, chest wall toxicity, including rib fracture, has been reported in the range of 6-46% (63). Tumors abutting central mediastinal structures are even more problematic. Haseltine reported 12% grade >3 toxicity in central or ultra-central tumors which rose to 30.7% when the tumor was <1cm from the proximal bronchial tree (64). Toxicities of concurrent chemo-radiation therapy (CRT) in locally advanced disease are similarly well described. In a meta-analysis of radiation toxicities in non-small cell lung cancer, Or and colleagues reported Grade >3 esophagitis and pneumonitis 22% and 11%, respectively, when concurrent chemotherapy was employed (65).

Management of tumor motion offers the potential to improve tumor control and reduce toxicity. Seppenwoolde and colleagues reported average amplitude of tumor motion was the greatest in the cranial-caudal direction for tumors located in the lower lobe and not attached to rigid structures (such as chest wall and vertebrae) compared to upper lobe and attached to rigid structures [12 ± 6 mm (SD) versus 2 ± 2 mm (SD)] (66).

Giaj-Levra and colleagues have suggested improved dose to cardiac structures resulting this motion management (67). Some have expressed concern over magnetic-field induced deviation of electron trajectories, creating hot or cold spots in relation to air-tissue interfaces. However, Raajimakers illustrated that with multiple and opposing beams, this concern is mitigated (68). Bainbridge and colleagues came to similar conclusions in a dosimetric study examining 10 plans in patients with locally advanced NSCLC and also suggested superior dose distributions could be achieved when MRI treatment employed smaller PTV margins which can be more easily achieved with real time tumor visualization (69).

### 6.3 Liver malignancies

Over the past four decades, the accumulated data collected from liver resections for patients with metastatic cancer demonstrated that the risk-benefit profile for hepatic resection shifted in favor of benefit (with long-term curative potential) for selected patients with primary colorectal tumors (70–72). This finding led to the proposed clinical state of oligometastases, whereby the anatomy and physiology may limit or concentrate metastases to a single or a limited number of organs that should be amenable to a curative therapeutic strategy (73). Interestingly, recent results from the randomized phase II SABR-COMET trial corroborated the idea that aggressive treatment of oligometastatic disease may improve overall survival (74). Although surgery remains the gold standard for patients diagnosed with primary or secondary liver tumors, not all patients are deemed to be surgical candidates. For nonsurgical candidates, radiofrequency ablation (RFA), trans-arterial chemoembolization (TACE), cryotherapy, trans-arterial radioembolization (TARE), and radiotherapy are frequently utilized alternative local treatment options, where the proper treatment selection relies on a multidisciplinary approach (75). The use of SBRT has increasingly been used in the management of liver metastasis and hepatic malignancies where many studies have reported their 2-year local control rates of ≥ 90%, comparable to other locoregional therapies (76). The control rates and survival for primary and secondary malignancies have somewhat shown to correlate with radiation dose (for example, cholangiocarcinoma [BED_10_ ≤ 80.5 Gy vs > 80.5 Gy], liver metastases [BED_10_ ≤ 100 Gy vs > 100Gy] (77, 78). Unfortunately, the liver, stomach, duodenum, bowel, and kidneys are radiosensitive organs and the radiation doses to these organs must be constrained (and the target dose reduced by proxy) to limit treatment related toxicities while treating liver lesions to ablative doses (79).

The use of stereotactic MR guided online adaptive radiation therapy for the treatment of primary and secondary liver malignancies represents a promising approach to improve the therapeutic ratio. This technique allows for better visualization of soft tissue, real-time tumor tracking, motion management (during the breathing cycle) *via* deep inspiration breath holding techniques (to allow for smaller target volumes), and adaptive planning (to improve target dosing while limiting dose to radiosensitive organs [that move with the respiratory cycle and change their juxtaposition daily] in line with recommended dose constraint guidelines) (74, 80). Although the normal organ constraints come from retrospective series, CT based planning (that assumes mobile structures remain in the same position throughout treatment), and animal studies, some have posited that radiation toxicity risks may be overestimated, the dose constraints too conservative, and the tumor unnecessarily underdosed (to meet the dose constraints) in patients treated with MRI guided adaptive radiotherapy (81). Thus, through a combination of raising the tolerance for normal organs at risk and reducing the margins delivered through MRI guidance, the overall dose to the tumor could be substantially increased or improved (82).

Recent and ongoing studies are working towards validating this hypothesis. In a phase I trial of MRI guided online adaptive radiation therapy (50Gy in 5 fractions, BED_10_ = 100Gy) for abdominal malignancies (20 patients with oligometastatic or unresectable primary liver cancers), Henke et al. demonstrated that adaptive planning allows for PTV dose escalation and/or simultaneous normal organ sparing compared to a non-adaptive SBRT approach (42, 83). Similarly, Rogowski et al. reported their early clinical experience of online adaptive MRI guided radiation therapy for liver tumors (84). Their retrospective series included treated patients with cholangiocarcinoma and various metastases (neuroendocrine tumors, colorectal carcinoma, sarcomas, and gastrointestinal stromal tumors). The median prescribed dose of BED_10_ = 84.4 Gy was delivered in 3 to 5 fractions. Adaptive planning was performed in 98% of fractions to improve PTV coverage and to reduce organ at risk constraint violations. After a median follow up of five months neither local failures nor ≥ grade 2 toxicities were observed. Ugurluer et al. reported their early experience in 21 patients with oligometastatic liver disease (85). The median dose delivered was 50 Gy in 5 fractions with 93 out of 111 fractions requiring re-optimization. All patients had either complete or partial response at their irradiated sites with an estimated 1-year overall survival of 93.3%. No ≥ grade 3 acute or late toxicities were observed. Padgett et al. reported their experience of 10 patients treated with MRI guided online adaptive SBRT for liver tumors (86). With a prescription dose range between 27-50 gray in 3-5 fractions and the daily utilization of adaptive planning, they observed significantly reduced PTV coverage for 32 out of 47 (68%) fractions and organ at risk constraint violations in 5 out of 23 (22%) fractions prior re-optimization. They concluded that online adaptive MR guided SBRT of liver tumors using daily re-optimization resulted in better target conformality, coverage, and organ at risk sparing compared with non-adaptive SBRT. Finally, in a prospective phase I trial van Dams et al. reported their outcomes after MRI-guided SBRT treatment of 20 patients with a mix of primary (8) and secondary (12) liver tumors (87). With a median follow-up of 18.9 months, they reported a 2-year local control rate of 79.6% utilizing a median dose of 54Gy in 3 fractions. Interestingly, they observed a local control difference between single vs multiple lesions and a BED_10_ < 100Gy vs BED_10_ ≥ 100Gy.

The ongoing MAESTRO randomized controlled phase 2 trial is testing the non-inferiority of MRI guided adaptive radiation versus ITV-based SBRT for hepatic metastases for hepatobiliary and gastrointestinal ≥ grade 3 toxicities (88). The secondary outcomes include local regional and distant tumor control, progression free-survival, overall survival, and the possibility of increase of BED using MRI guided radiotherapy if the BED is limited with ITV base SBRT. The results of this trial will further define whether MRI guided adaptive radiotherapy provides for an improved therapeutic ratio as compared to standard ITV-based SBRT for liver lesions.

### 6.4 Pancreatic cancer

The role of radiation therapy for pancreatic ductal adenocarcinoma (PDAC) remains unclear. Recently reported clinical trials have not shown any significant improvement in overall survival in the localized setting (89, 90). It has been hypothesized that one of the reasons for the limited efficacy seen in the recent trials with pancreatic radiotherapy has been the delivery of non-ablative radiation doses to the pancreatic tumor target. A role for radiation dose escalation in PDAC is supported by other studies which use higher ablative doses, showing improved local control and potentially survival (91–93).

The major challenge in pancreatic radiotherapy is delivering significant radiation doses to the pancreatic target without causing significant toxicity. In particular, the stomach, the duodenum, bowel, and other nearby radiosensitive organs often receive significant collateral radiation doses. The significant toxicities attributable to pancreatic radiation treatments highlight the limitations of conventional techniques (94–97). Applying MRgRT shows great promise in overcoming the clinical challenges of organ motion and daily anatomic changes particular to pancreatic radiotherapy.

Stereotactic MR-guided on-table Adaptive Radiation Therapy (SMART) is an MRgRT application designed to account for inter-fractional anatomic changes. It utilizes the MRI scans acquired both before and continuously during treatment delivery to account for intra-fractional motion management to deliver ablative radiation doses. A retrospective study of 5-fraction SMART in locally advanced PDAC showed promising efficacy and safety (92). More recently, a multi-institutional prospective Phase 2 trial of SMART in localized pancreatic cancer completed accrual and presented early results, showing promising efficacy and toxicity outcomes (98). Pancreatic cancer treatment may be one of the most apparent direct applications of MRgRT with several Phase 3 clinical trials in the development phase that plan to test whether ablative pancreatic radiotherapy may improve overall survival in the locally advanced PDAC setting. There is eager anticipation to gather further prospective clinical data on the role of ablative pancreatic radiotherapy made feasible by adaptive image guided radiotherapy.

### 6.5 Breast cancer

In early-stage breast cancer management, there is significant momentum towards de-escalating intensity of treatment. Local recurrence rates in early stage disease have decreased over time and are reported to be less than 5% over 10 years of follow up in recent clinical trials (99–101). Nevertheless, fear of recurrence after treatment and long-term toxicities associated with treatment remain primary issues in survivorship, and local and distal breast cancer recurrence risk remains a concern for patients even 20 years or more after treatment for early stage breast cancer (102).

De-escalation in breast radiotherapy has emphasized treating smaller target volumes and prioritizing avoiding the nearby heart and lungs, based on the improvements in image-guided radiotherapy technologies. Several large Phase 3 trials have demonstrated good efficacy and toxicity of external beam partial breast irradiation (PBI) when compared with whole breast radiotherapy (99, 101). For external beam PBI, a current standard is daily CT-based image guidance. The capability of on-table MRI guidance, adaptive planning, and delivery with the prone technique has several particular potential advantages over CT-based imaging.

MR imaging is superior in delineating soft tissue contrast in the breast, allowing for more accurate surgical cavity and target volume delineation. Intra-fractional MR-guided imaging and decreased chest wall excursion with the prone breast setup can further account for organ motion and decrease targeting uncertainty (103). MRI guidance would potentially allow CTV and PTV margins to be reduced. With potentially smaller treatment volumes, MRgRT may decrease toxicities associated with breast radiotherapy (104).

The versatility of daily adaptive imaging, contouring, planning, and radiation delivery process can help mitigate the challenges of daily setup uncertainties for breast radiotherapy. The surgical cavity can be difficult to delineate even with surgical clip or marker placement within the surgical bed, a practice not universally followed by breast surgeons. When surgical clips are placed to mark the cavity, they can migrate from their initial position within the breast over time. It is known that the surgical cavity can change over the time it takes to deliver a course of breast radiotherapy (105). Significant topological and volume changes in the breast can occur during the course of radiation treatment (106). Nearby organs at risk can also change in relative position on a day-by-day basis. Of particularly clinical concern, cardiac positioning can vary considerably relative to the bony anatomy and other anatomic landmarks used for patient positioning (107). Even with prone positioning, the left anterior descending coronary artery can receive significant radiation dose if daily imaging guidance is not utilized (108). For those patients where positional setup uncertainty is significant, similar to the standard for PBI, it is recommended that more frequent image-guidance be utilized during breast radiotherapy, such as daily cone-beam CTs (109). A technique of combining MRgRT with prone breast irradiation, which we coin Precision Prone Irradiation (PPI) enables greater flexibility and potentially more accurate treatment delivery (110).

As explained above, CT-based breast treatment planning often requires frequent or daily cone beam CT imaging for setup verification. For patients with high aversion to the radiation exposure from daily CT scans or tattoo marking, MRgRT offers the opportunity to potentially bypass the CT simulation, permanent skin tattooing, or regular cone beam CT image verifications which have become standard. The potential promise of smaller radiation treatment volumes through the PPI technique and the significance of any such advantages will have to be established through prospective clinical trials.

### 6.6 Central nervous system tumors

In the CNS, for both primary parenchymal brain tumors and for brain metastases, identifying post-planning changes in target volumes that have occurred since their delineation permits more accurate delineation of target volumes at the time that treatment is actually being delivered. Changes in the position of both target volumes and normal tissues at risk of injury from irradiation have been documented to be increasingly likely to occur with longer elapsed time since a dedicated planning study (111, 112).

For patients with intrinsic brain tumors where the operative bed is part of the target volume, the gradual resolution of mass effect after craniotomy will present a very different substrate for contouring (and planning) depending on when the imaging for planning irradiation is performed. Changes resulting from the craniotomy may continue to occur during a several month period after surgery is performed—during the time that fractionated radiotherapy will be delivered.

Unfortunately, the competing capabilities of a hybrid MR-Linac hinder both the imaging and treatment delivery capabilities relative to dedicated machines. Specialized imaging assessments such as chemical exchange saturation transfer imaging and diffusion weighted imaging have been performed on a 1.5T MRI-Linac (113, 114). Many specialized pulse sequences have not yet been demonstrated on an MRI-Linac, and lower field strength scanners may never be able to perform many specialized sequences.

Also, it may not be cost-effective to use the MRI capabilities of a MRI-Linac to perform specialized assessments when treatment throughput is an important criterion of successful implementation of this technology. Magnetic resonance fingerprinting, still in its relative infancy, allows the simultaneous measurement of multiple tissue properties in a single, time-efficient manner, and may permit serial assessments of responses to radiation treatment to be gathered during the re-planning guided by anatomic information (115, 116). It remains to be confirmed if serial short acquisitions during daily treatment can provide oncologically important information to help with guiding treatment recommendations for patients with CNS malignancies.

It may be argued that for radiosurgical treatments, the short duration of treatment delivery (1-5 sessions) will prevent significant shifts in the location and conformation of target volumes and contiguously located critical normal tissues. A single adaptation on the first day of treatment may be all that is needed, but it is also possible that changes in the target volumes consequent to the treatments already delivered will require changes in one or more additional fractions (117). There is no prospectively acquired data from daily MR imaging during a course of hypofractionated radiosurgery that might adapt current treatment recommendations for brain metastasis or benign tumor (meningioma, schwannoma, etc.) treatments, where a single treatment plan generated and checked before the start of therapy is used for all delivered fractions.

Investigators at Sunnybrook Medical Center in Toronto have conducted a prospective study of sequential MRI scans on a 1.5T MR-LINAC that were done on the first day, 11^th^ day, and 21^st^ day of partial brain radiotherapy in a 6 week course of treatment. Both the locations and sizes of the target volume locations were evaluated. The gross target volume decreased over the course of therapy in most patients with a median volume decrease from 18.4 cm^3^ on day 1 to 14.7 cm^3^ on day 11, and 13.7 cm^3^ on day 21. The intracranial position of the target volume changed during the course of therapy as well. Migrations of >0.5 cm in the target volumes were seen in 54% of patients by the beginning of the 3^rd^ week of radiotherapy (day 11 imaging), and 58% of patients by the beginning of the 5^th^ week of radiotherapy (day 21 imaging) (118). The large margins used for radiotherapy of malignant gliomas may lower the probability of a complete geographic miss, but being able to decrease target volume margins while avoiding geographic misses would potentially benefit many patients receiving partial brain radiotherapy by exposing less brain to high-dose irradiation.

Over a multiple-week course of radiation therapy, as is commonly delivered for an intrinsic low- or high-grade glioma, or for a pituitary neuroendocrine tumor, craniopharyngioma, or meningioma located close to the anterior visual pathways, there also may be changes in the locations of the target volume and normal tissues at risk for morbidity when compared to an MRI scan performed 1-2 weeks or more prior to initiation of radiotherapy. Indeed, for craniopharyngioma treatments, it is advised to have an MRI scan done part-way through treatment to ensure that there has been no change in the target volume that would necessitate re-planning to reflect the new anatomic realities (119). Finally, there are relatively mobile targets within the CNS (optic nerve sheath meningioma) where a patient’s eye position on the treatment planning MRI and simulation CT scan may not be matched for any of the 5+ weeks of daily radiotherapy. Having a daily confirmation of the position of the target would perhaps improve the therapeutic ratio for this particular tumor.

MRgRT may be particularly valuable for soft-tissue imaging in the presence of surgically implanted devices such as spinal fixation hardware in patients who have had surgical stabilization of their spines and require irradiation for control of metastatic cancer. The low field strength permits visualization of the bony and soft-tissue anatomy in the area where the radiation is required. Conventional 1.5 or 3T imaging introduces artifact from the surgical stabilization devices, and the presence of artifact from the hardware also degrades CT imaging so that the spinal cord’s exact position may only be determined by a CT myelogram (120). Dosimetric studies suggest a potential impact for the MRI-Linac in spinal irradiation or re-irradiation (121–123).

## 7 Future developments

In this section, we will highlight what are some of the most exciting and evident developments emerging in MRgRT. As we described above, adaptive MRgRT treatment currently require significant time dedicated by the clinical team members. One development which has emerged in the past year is an upgrade to the MRI-Linac system that allows for parallel workflow processes in the treatment planning process. These upgrades should allow multiple users to work on the plan simultaneously, thereby reducing the total time needed for adapted fractions. Two other potential high impact developments that we will describe in more detail are the intense ongoing research in generating synthetic CT (sCT) images and in radiomics.

### 7.1 Synthetic CT

**Figure 6:**
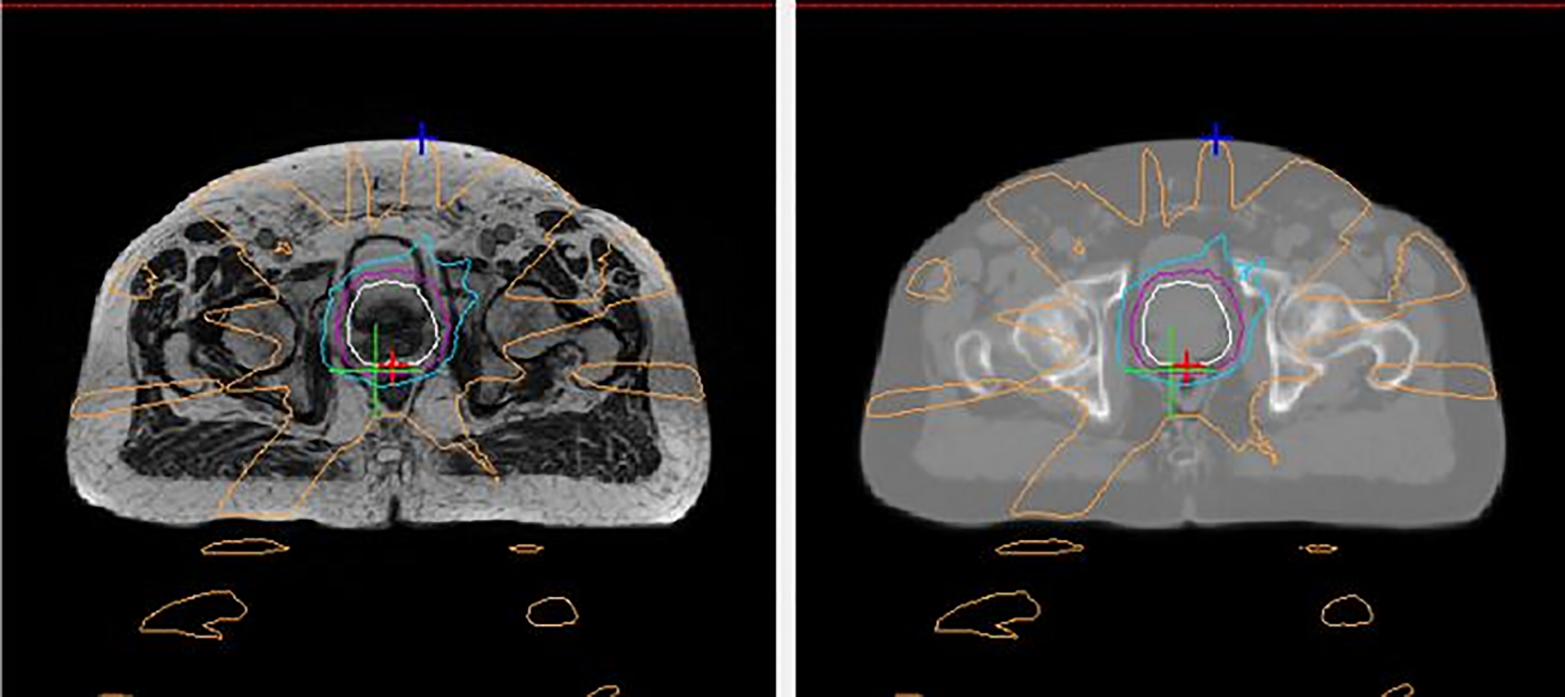
An example of a generated synthetic-CT (sCT) for prostate radiotherapy. The left panel shows an axial MR image acquired with a MRI-Linac during a MRI simulation. The right panel shows the corresponding sCT generated based on that MRI image by converting MR intensities into Hounsfield Units.

The workflow for treatment on a MRI-Linac is a two-step process: one based on MR imaging acquisition and other based on CT imaging acquisition. The disadvantage of the MRI simulation is the lack of electronic density tissue information that are fundamental in RT planning to calculate the dose distribution. These essential data are typically derived from CT based on Hounsfield Units (HU) and cannot be obtained directly from MR images. Moreover, this crucial aspect is necessary both to start treatment planning and during treatment to evaluate adaptive re-planning. To overcome this limit, several solutions have been proposed to convert MR intensities in HU, generating synthetic-CT images (**Figure 6**) (124).

Three main domains summarize these solutions: bulk density, atlas-based, and machine learning (ML) methods. Bulk density is the least sophisticated and time consuming method. It consists of grouping structures with similar density and attributing a homogeneous electronic value without taking into account the tissue heterogeneity. The atlas-based methods require co-registration of CT with MR images based on library collections. Although it is possible to evaluate tissue heterogeneity, the co-registration and the different position/anatomy of the patient is an important limitation of the method. Finally, ML and deep learning approaches are the most promising and most recently investigated methods that allow fast and accurate sCT (125). In 2017, Han proposed a novel deep convolutional neural network method for sCT generation, showing that it is able to produce accurate sCT in real time and opening the way for future developments (126). The main DL architectures used for sCT generation are the U-Net and the Generative Adversarial Networks (GAN) (127). A narrow restriction is represented by the ability of these models to adequately respond to considerable anatomical variations. While the central nervous system is easily reproducible and predictable, anatomic regions such as the head and neck, the pelvis, and the abdomen are examples of extreme variability due to physiological changes. Today, the search for a standardized, reliable, and applicable model for various anatomical and clinical scenarios remains a great challenge.

### 7.2 Radiomics

Radiomics is often defined as the application of quantitative imaging analysis to convert images to higher dimensional data and the subsequent mining of these data for improved decision support when integrated with clinical data (128). Radiomics analysis could potentially predict tumor response based not just on morphological criteria, according to Response Evaluation Criteria in Solid Tumor (RECIST), but also through integration of biological and functional data to implement predictive-prognostic models of survival (129–131). In this field, MRI has potential beyond other imaging modalities (e.g. CT and PET) by providing morphological and functional data together (132, 133). In particular, dynamic-contrast enhanced (DCE) images can describe vascularization and apparent diffusion coefficients (ADC) derived by diffusion weighted imaging (DWI) can quantify cellularity, applications with great potential if contrast is administered before MRgRT treatments. These biologically relevant data have a potential key role to predict treatment response while taking into account the ability to quantitatively describe cell metabolism and death. Furthermore, combination with morphological and structural data could lead to an accurate description of the tumor microenvironment (134). The versatility of the MRI-Linac technology opens the possibility to collect this biologic data while imaging and treating the patient.

The availability of repeat MRI imaging during treatment also opens the possibility of another promising treatment response analysis: delta radiomics. The rationale behind delta radiomics is that the combined analysis of images acquired before, during and after treatment can provide a more complete description of tumor behavior, including sensitivity of the individual patient to a specific treatment (135). Considering the clinical impact of immunotherapy and precision medicine, a current challenge is the ability to evaluate changes in the microenvironment induced by the immune response or targeted therapy, which may be possible by combining multiparametric MR images (136–138).

## 8 Conclusion

MRgRT is a transformative radiotherapy technology that is having significant impact in clinical radiation oncology. This novel technology allows the clinical team to improve on three staples of the radiation treatment process: therapy guidance, treatment verification, and delivery control. In inter-fractional and intra-fractional management, MRgRT offers several advantages over current standard radiotherapy technologies. However, these advantages are counterbalanced by increased costs, increased resource and time allocation, and other practical limitations. This pattern is similar to previous disruptive radiotherapy technologies where further refinements and clearer clinical roles enabled widespread adoption. The utilization and capabilities of the MRI-Linac are already expanding in a wide range of clinical disease sites. Future advances will smoothen the MRgRT treatment planning and delivery process. Advances in MR imaging, radiomics, and advances in artificial learning/machine learning will further leverage this technology’s clinical potential. We expect the utilization of the MRgRT platform to grow and reshape the radiation oncology clinic in the coming decades.

## Author contributions

All authors listed have made a substantial, direct, and intellectual contribution to the work, and approved it for publication.
